# Sex differences in risk-based decision making in adolescents with conduct disorder

**DOI:** 10.1007/s00787-017-1024-9

**Published:** 2017-07-07

**Authors:** Justina Sidlauskaite, Karen González-Madruga, Areti Smaragdi, Roberta Riccelli, Ignazio Puzzo, Molly Batchelor, Harriet Cornwell, Luke Clark, Edmund J. S. Sonuga-Barke, Graeme Fairchild

**Affiliations:** 1 Academic Unit of Psychology, University of Southampton, Building 44, Southampton, UK; 2 Department of Psychology, University of British Columbia, Vancouver, Canada; 3 Department of Child and Adolescent Psychiatry, Institute of Psychiatry, Psychology and Neuroscience, King’s College London, London, UK; 4 Department of Psychology, University of Bath, Bath, UK

**Keywords:** Conduct disorder, Antisocial behaviour, Sex differences, Decision making, Risk, Reward

## Abstract

Altered decision making processes and excessive risk-seeking behaviours are key features of conduct disorder (CD). Previous studies have provided compelling evidence of abnormally increased preference for risky options, higher sensitivity to rewards, as well as blunted responsiveness to aversive outcomes in adolescents with CD. However, most studies published to date have focused on males only; thus, it is not known whether females with CD show similar alterations in decision making. The current study investigated potential sex differences in decision making and risk-seeking behaviours in adolescents with CD. Forty-nine adolescents with CD (23 females) and 51 control subjects (27 females), aged 11-18 years, performed a computerised task assessing decision making under risk—the Risky Choice Task. Participants made a series of decisions between two gamble options that varied in terms of their expected values and probability of gains and losses. This enabled the participants’ risk preferences to be determined. Taking the sample as a whole, adolescents with CD exhibited increased risk-seeking behaviours compared to healthy controls. However, we found a trend towards a sex-by-group interaction, suggesting that these effects may vary by sex. Follow-up analyses showed that males with CD made significantly more risky choices than their typically developing counterparts, while females with CD did not differ from typically developing females in their risk-seeking behaviours. Our results provide preliminary evidence that sex may moderate the relationship between CD and alterations in risk attitudes and reward processing, indicating that there may be sex differences in the developmental pathways and neuropsychological deficits that lead to CD.

## Introduction

Decision making plays a pivotal role in everyday functioning. From a neuropsychological perspective, decision making encompasses a variety of distinct processes [1], and is an important determinant of an individual’s successes and failures in life [2]. Efficient decision making requires constant updating of value representations and the evaluation of potential outcomes, for instance, estimates of the magnitude of potential gains and losses and the respective probabilities of their delivery are integrated to yield the expected value of that choice [3]. Although this implies that the option with the highest expected value should always be chosen, decision making in human participants is strongly influenced by psychological factors affecting subjective value assignment [4, 5]. One such factor is the propensity for taking risks when outcomes are uncertain. There are also individual differences—some individuals are more prone to risk-seeking behaviours, while others may be significantly risk averse [6].

Abnormalities in decision making processes have been implicated in many childhood and adolescent psychiatric conditions and neurodevelopmental disorders. Conduct disorder (CD), a pervasive and persistent pattern of antisocial behaviour that emerges in childhood or adolescence [7], is associated with decision making deficits which are related to both excessive risk-seeking and difficulties in learning from aversive outcomes [2, 8]. This preference for risk and insensitivity to punishment may contribute to disruptive behaviours typical of CD, e.g., reckless choices that place the individual themselves or others in physical danger, problematic substance use, and continued involvement in criminal activity, despite punishment within the youth justice system or even incarceration [9, 10]. The existing studies in this field provide compelling evidence for CD-related abnormalities in decision making and risk taking behaviours. In particular, previous behavioural and psychophysiological findings indicate an increased preference for risky options, as well as higher sensitivity to rewards and blunted responsiveness to aversive outcomes, in individuals with CD or related disruptive behaviour disorders such as oppositional defiant disorder [8, 11–13].

It is striking, however, that despite compelling evidence from normative studies showing that typically developing males have an increased propensity towards risk taking compared to females [14–18], the vast majority of studies investigating decision making in CD or oppositional defiant disorder have included male participants only (e.g., [8, 12, 13]). The few existing studies investigating potential sex differences in CD have provided support for differential developmental profiles for male and female CD, manifested by different behavioural patterns (e.g., [19]), as well as sex-specific neural alterations (e.g., [20–22]). Therefore, given the previously identified sex differences in risky decision making in normative populations, and our currently limited understanding of the neuropsychological profile associated with CD in females, the present study examined the potential role of sex as a moderator of (risky) decision making in CD. We investigated risky decision making in male and female adolescents with CD compared with age- and sex-matched typically developing controls, and tested whether the relationship between CD and risky decision making differs according to sex.

To achieve this, we employed a modified version of the Risky Choice Task [23], which is an experimental paradigm assessing decision making under risk. In each trial, participants choose between two possible gambles that vary in the relative probability of gaining and losing points, as well as the magnitude of the potential gains and losses. On each trial, a control gamble offers a 50–50 chance of winning or losing a small number of points. This option is pitted against an experimental or ‘risky’ gamble, which varies across trials in terms of the relative probabilities and magnitudes of potential gains and losses. The experimental gamble is always riskier (i.e., associated with greater outcome variance) than the control gamble, but in some trials the experimental gamble is favourable, whereas in other trials it is unfavourable. The systematic manipulation of the probabilities and magnitudes of gains and losses in this task provides a way to examine the participants’ decision making in the face of different levels of risk and possible gains and losses. In addition, the Risky Choice Task includes two conditions designed specifically to evaluate decision making under conditions involving only losses or only gains, termed the ‘reflection effect’ [24, 25]. The ‘reflection effect’ denotes the contrasting risk preferences, which people exhibit for uncertain choices based on whether the outcome is framed as a gain or a loss. On the gains-only trials, participants choose between a certain gain of moderate value (40 points) and a gamble offering a 50–50 chance of a large gain (80 points) or no gain (zero points). Conversely, the losses-only trials involve a choice between a certain moderate loss (−40) and a gamble offering a 50–50 chance of a large loss (−80) or avoiding a loss altogether (zero points). In each case, the two choices are matched in terms of expected value, but healthy participants are typically risk-seeking on the losses-only trials, and risk averse on the gains-only trials. In other words, one is generally risk averse when there is an option of a certain gain, but risk-seeking when choosing between a certain loss and a risky chance of gain (see [24, 26–28]).

The Risky Choice Task has previously been applied to study risky decision making and sensitivity to punishing and/or rewarding outcomes in (child) psychiatric populations and has proved to be a valid instrument [8, 13, 23, 29, 30]. In relation to CD, the findings from studies employing this paradigm indicate increased risk taking and higher sensitivity to rewards or reduced sensitivity to punishments in males with this disorder [8, 31]. Hence, in the current experiment we expected a similar pattern of decision making in individuals with CD. In particular, we hypothesized that CD individuals would exhibit a tendency towards increased risk taking compared with typically developing control subjects. With regard to sex effects, we predicted that female participants would be more cautious in terms of decision making than male participants. Importantly, we expected to observe sex-by-group interactions in risk taking. Based on the evidence from the existing literature on males with CD (e.g., [8, 13]) and sex differences in decision making in normative populations (e.g., [14, 15, 17]), we predicted that increases in risk taking would be most pronounced in males with CD.

## Methods

### Participants

The participants in this study were part of the sample recruited for a multisite European Commission-funded research project entitled ‘The Neurobiology and Treatment of Adolescent Female Conduct Disorder’ (FemNAT-CD; http://www.femnat-cd.eu/). This large-scale research project aims to investigate CD in females and examine potential sex differences in the causes and correlates of CD.

Forty-nine adolescents with CD (23 females) and 51 typically developing control participants (27 females) aged 11-18 years participated in the current study. Typically developing control subjects were recruited through mainstream primary and secondary schools and further education colleges via mail-outs of information packs, invited presentations in school assemblies, and e-mails to parents circulated via school distribution lists. Participants with CD were also recruited from mainstream schools and colleges, but primarily from specialist education centres and pupil referral units for children with emotional and behavioural difficulties and local Youth Offending Services in the Hampshire region of the UK.

The current study was conducted at the University of Southampton and was approved by the University Ethics Committee and the National Health Service Research Ethics Committee. Written informed consent or assent was obtained from each participant and their parent or carer, prior to their participation in the study. Participants received a monetary payment for taking part. If the participant was aged 16 years or above, informed consent was only obtained from him/her and not from the parent or carer. All participants were informed that they were free to withdraw from the study at any time.

Diagnostic interviews were completed with all the participants and the majority of parents or carers, using the Schedule for Affective Disorders and Schizophrenia for School-Age Children-Present and Lifetime Version (K-SADS-PL) [32], to assess current and lifetime psychopathology. This semi-structured interview is based on DSM-IV diagnostic criteria [33]. In order for a research diagnosis of CD to be given, at least three symptoms were required to be present in the past 12 months and at least one symptom in the past six months [33]. All interviewers received extensive training and were shadowed by highly experienced staff members before undertaking K-SADS-PL interviews independently. The inter-rater reliability values for CD, ADHD, ODD and depression based on the K-SADS-PL were very high (Cohen’s kappas ranging between 0.84 and 0.95, so almost perfect agreement between raters).

Exclusion criteria for participation included having an IQ <70, as estimated using the two-subtest version of the Wechsler Abbreviated Scale of Intelligence [34]: a formal diagnosis of an autism spectrum disorder or a neurodevelopmental syndrome, psychosis, neurological disorders and/or having experienced a serious head injury. Further exclusion criteria, applicable only to the control participants, were a current diagnosis of an Axis I disorder or past diagnoses of CD, ODD, or ADHD. Psychopathic and CU traits were assessed using the self-report Youth Psychopathic traits Inventory (YPI; [35]). YPI has been proven to be a valid and reliable self-report instrument to assess psychopathic traits [36–39].

### Decision making task

A modified version of the Risky Choice Task was employed [23], which involves the presentation of roulette wheels rather than shaded bars to depict potential gains and losses and their respective probabilities, in an attempt to make the decision the subject is making easier to understand and the task more accessible and enjoyable. In addition, the fact that a ticker spins around, eventually landing on a segment of the wheel, makes the task more exciting and emotionally laden than the original version of the task that was created by Rogers and colleagues (2003).

**Fig. 1:**
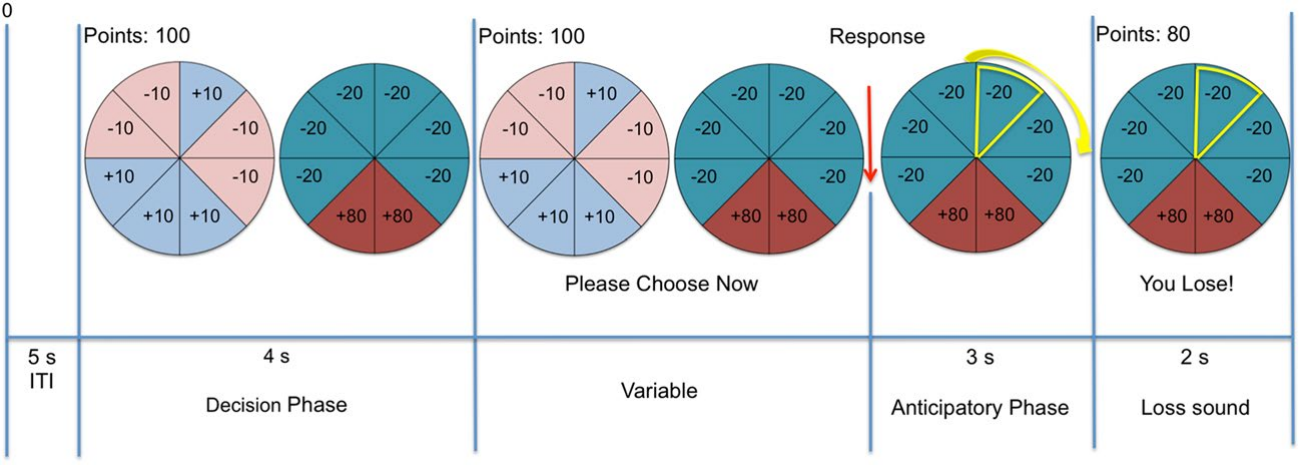
A schematic representation of one trial of the modified Risky Choice Task used in this study. The control gamble is shown on the *left* side and has an expected value of 0 (0.5 × 10 + 0.5 × −10). The experimental gamble is shown on the right side and has an expected value of +5 (0.75 × −20 + 0.25 × 80). The difference in expected values (delta) between the gambles is therefore +5, so the experimental gamble is more favourable than the control gamble in this instance. Following the participant’s response (i.e., choice of one gamble), the *yellow highlight spins* around the wheel, gradually slowing until it stops on one of the wedges. The participant receives visual and auditory feedback, as well as being presented with the updated points total after each trial

At the beginning of each trial, two roulette wheel gambles were presented on the computer screen, depicting the potential wins and losses available in points and the relative probability of each outcome (see Fig. 1). Participants were instructed to choose one of the wheels on each trial. There were a total of 10 trial types—8 involved a choice between an identical control gamble and a varying experimental gamble, with the two options on each trial always differing in their expected values (EVs). On the other two framing trial types, the two options differed in outcome variance but were matched for expected value. On the 8 trial types involving the control gamble, this was a ‘safe’ option offering a 50% probability of winning 10 points and a 50% chance of losing 10 points (it therefore had an EV of 0). The other wheel was the experimental or ‘risky’ gamble; it varied in terms of the probability of winning points (either high or low, i.e., 75 vs. 25%), the magnitude of expected gains (either large or small, i.e., 80 vs. 20), and the magnitude of the possible losses (either large or small, i.e., 80 vs. 20), giving rise to 8 different combinations. On these trials, the differences (delta) between the expected values (∆EVs) of the two wheels were as follows: −55, −40, −10, −5, 5, 10, 40 and 55, reflecting a shift from the experimental gamble being highly unfavourable (negative EV) to highly favourable (positive EV; see Table 1 for further information about the specific trial types). In addition, there were two ‘reflection’ trials. Typically developing individuals are risk averse (i.e., show a preference for certain gains) in positively framed trials, whereas they tend to be risk-seeking (i.e., take a chance to avoid a certain loss) in negatively framed trials [24, 28]. In the current task, the gains-only trials presented a choice between a guaranteed gain of 40 points and a second wheel that yielded a 50% chance of gaining 80 points or a 50% chance of receiving 0 points. These options are mathematically equivalent, but the variance in outcome values is higher in the latter case. In the losses-only trials, one wheel offers a certain loss of 40 points while the second wheel offers the chance of either losing 80 or 0 points. During the course of the experiment, the 10 trial types were each repeated 4 times and pseudo-randomly intermixed across four blocks. The position of control and experimental gamble wheels on the screen (i.e., right–left) was randomized. The decision making phase included an imposed waiting period of 4 s before the participants could make their response. At the beginning of each block, participants were given an endowment of 100 points and instructed to try to gain as many points as possible. At the end of each trial, the participant received visual and auditory feedback and was presented with the updated points total. A schematic representation of the task is displayed in Fig. 1.

**Table 1:** Delta expected values across the ten trial types used in the risky choice task

Risky wheel			Safe wheel			Difference in expected value (ΔEV) between wheels	Trial number
Pr (gain)	Gain	Loss	Pr (gain)	Gain	Loss		
0.25	20	−80	0.50	10	−10	−55	2
0.25	80	−80	0.50	10	−10	−40	4
0.25	20	−20	0.50	10	−10	−10	6
0.75	20	−80	0.50	10	−10	−5	3
0.25	80	−20	0.50	10	−10	+5	0
0.75	20	−20	0.50	10	−10	+10	7
0.75	80	−80	0.50	10	−10	+40	5
0.75	80	−20	0.50	10	−10	+55	1
0.50	0	−80	0.00	0	−40	0 (− frame)	8
0.50	80	0	1.00	40	0	0 (+ frame)	9

− and + frame indicate the negative and positive framing trials

*Pr* probability

### Statistical analysis

Data on participant demographic, clinical and personality characteristics were analysed using Chi-square tests and two-way ANOVAs with Sex and Group as between-subjects factors. Task data were analysed using repeated-measures ANOVA with Trial Type (i.e., ΔEV of a trial or gain/loss for ‘reflection-trials') as a within-subjects factor and Group (CD, controls) and Sex (male, female) as between-subjects factors. The dependent variable was the percentage of risky choices made in each trial type, i.e., choice of the experimental gamble on the (1) 8 ΔEV trials and (2) on the ‘reflection-effect' trials (i.e., loss and gain trials). In the analysis of potential confounding factors, we controlled for IQ and ADHD symptoms. Hence, the ANCOVA analyses included Trial Type (i.e., ΔEV of a trial or gain/loss for 'reflection-trials') as a within-subjects factor and Group (CD, controls) and Sex (male, female) as between-subjects factors with IQ and ADHD scores as covariates. Correlation analyses using Pearson’s correlation coefficient were performed between CD symptoms and participants’ risk preferences on the 8 ΔEV trials and reflection trials. For all analyses, we report the resulting F-statistic, the associated *p* value, and partial eta-squared values ($\eta_{\text{p}}^{2}$; small = 0.01; medium = 0.06; large = 0.14) as an estimate of effect size [40, 41]. All statistical analyses, including *observed power* calculations, were performed using the IBM SPSS statistics package (version 24; IBM, Armonk, NY).

## Results

### Demographic characteristics

The groups did not differ in age (*p* = 0.202) or sex composition [there were no group differences in the proportion of males and females (*χ* ^2^(1) = 0.36 *p* = 0.548)]. The CD group had a significantly lower mean IQ than the control group, as is typical in studies of this type, but there was no Sex-by-Group interaction for IQ (see Table 2). As expected, the CD group reported significantly more CD and ADHD symptoms than the control group, and also scored higher in total psychopathic traits and CU traits, but there were no significant Sex-by-Group interactions for these measures (Table 2).

**Table 2:** Demographic, personality and clinical characteristics of the sample

Measure	Controls				CD				*p*
	Male (*n* = 24)		Female (*n* = 27)		Male (*n* = 26)		Female (*n* = 23)		
	Mean	SD	Mean	SD	Mean	SD	Mean	SD	
Age (years)	14.79	2.18	15.51	1.94	15.76	2.15	15.60	1.72	ns
Estimated IQ	101.33	11.77	95.85	14.22	90.88	9.78	92.04	14.07	0.006* (controls > CD)
YPI-total	97.83	12.98	90.00	15.31	106.88	24.68	104.86	19.84	0.002* (CD > controls)
YPI-CU	22.54	3.71	18.59	3.69	25.76	5.89	19.95	4.46	0.013* (CD > controls) <0.001** (male > female)
CD Symptoms	0.16	0.38	0.11	0.32	4.92	3.07	3.65	2.14	<0.001* (CD > controls)
ADHD Symptoms	0.04	0.20	0.22	0.69	4.69	4.73	4.08	4.02	<0.001* (CD > controls)
					Comorbid conditions			*N*	
					Depression			3	
					Substance abuse			4	
					Psychoactive medication			*N*	
					Concerta			2	
					Strattera			2	
					Venlafaxine			1	
					CD onset type^^^			*N*	
					Childhood onset			22	
					Adolescence onset			12	

*CD* conduct disorder, *SD* standard deviation, *IQ* intelligence quotient, *YPI-total* total score on the Youth Psychopathic traits Inventory, *YPI-CU* callous-unemotional scale score on the Youth Psychopathic traits Inventory, *CD* symptoms number of conduct disorder symptoms from the K-SADS-PL, *ADHD* attention-deficit/hyperactivity disorder, *ns* no significant effects

* Significant effect of Group

** Significant effect of Sex

^^^Age-of-onset data were unavailable for 15 subjects

### Risky choice task performance

**Fig. 2:**
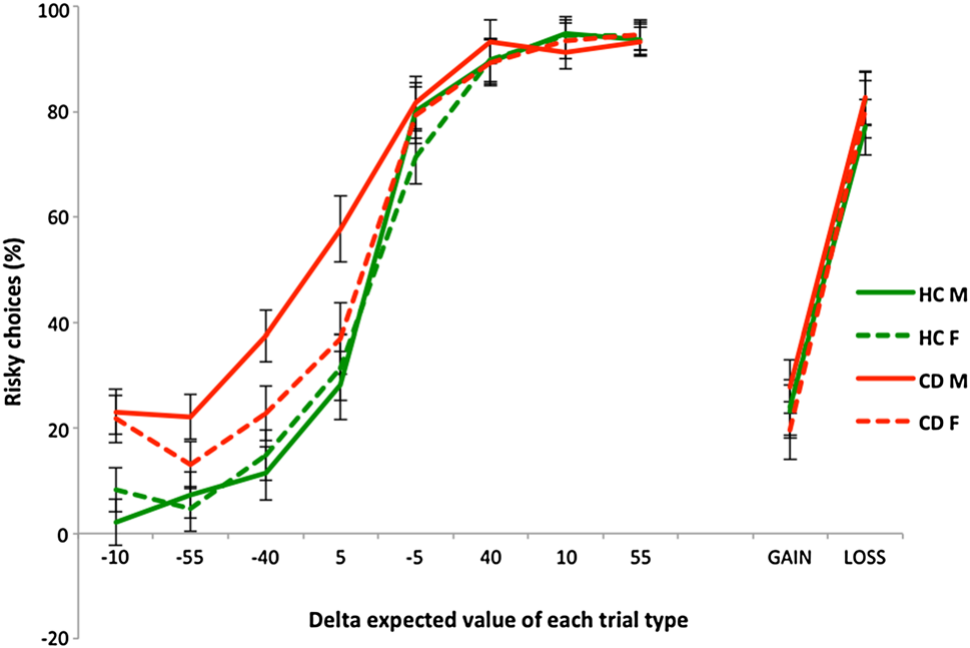
Mean percentage of risky choices according to expected value, by group. *Error bars* represent ±1 standard error of the mean. *HCM* healthy control males, *HCF* healthy control females, *CDM* conduct disorder males, *CDF* conduct disorder females. The gain and loss trials show the data for the positive and negative reflection trials, respectively

When considering the main 8 ΔEV trials, there were main effects of Trial Type [*F*(4.21, 405.04) = 294.82, *p* < 0.001; $\eta_{\text{p}}^{2}$ = 0.754] and Group [*F*(1, 96) = 13.25, *p* < 0.001; $\eta_{\text{p}}^{2}$ = 0.121] on the number of risky choices made, as well as a significant Trial Type × Group interaction [*F*(4.21, 405.04) = 4.46, *p* = 0.001; $\eta_{\text{p}}^{2}$ = 0.044]. Hence, individuals with CD tended to choose the risky option more frequently and this group difference was most evident (as significant simple effects) on trials with ΔEVs of −55, −40, −10, and +5 (Fig. 2). We did not find a significant main effect of Sex [*F*(1, 96) = 2.74, *p* = 0.101; $\eta_{\text{p}}^{2}$ = 0.028], a significant Trial Type × Sex interaction [*F*(4.21, 405.04) = 0.98, *p* = 0.418; $\eta_{\text{p}}^{2}$ = 0.010], or a significant Trial Type × Sex × Group interaction [*F*(4.21, 405.04) = 1.52, *p* = 0.191; $\eta_{\text{p}}^{2}$ = 0.016]. However, we did observe a trend towards a significant Group × Sex interaction [*F*(1, 96) = 3.13, *p* = 0.080; $\eta_{\text{p}}^{2}$ = 0.032; *observed power* = 0.418]. Comparisons within the male and female samples showed that this trend-level interaction was driven by males with CD making significantly more risky choices than healthy males [*F*(1, 48) = 18.51, *p* < 0.001; $\eta_{\text{p}}^{2}$ = 0.278], with a large effect size, whereas females with CD did not significantly differ from healthy females in terms of the number of risky choices made [*F*(1, 48) = 1.44, *p* = 0.235; $\eta_{\text{p}}^{2}$ = 0.029].

The reflection trials were examined in a separate model with Condition (*gain*, *loss*) as a within-subjects factor and Group and Sex as between-subjects factors. There was a main effect of Condition [F(1, 83) = 221.41, *p* < 0.001; $\eta_{\text{p}}^{2}$ = 0.69], with both the CD and control groups choosing the risky option more frequently in the *losses*-*only* condition than the *gains*-*only* condition, consistent with the classic reflection effect observed in normative adult populations. However, there was no main effect of Group [*F*(1, 96) = 0.079, *p* = 0.779, $\eta_{\text{p}}^{2}$ = 0.001], no main effect of Sex [F(1, 96) = 0.18, *p* = 0.670, $\eta_{\text{p}}^{2}$ = 0.002], as well as no Group × Sex interaction [*F*(1, 96) = 0.13, *p* = 0.290; $\eta_{\text{p}}^{2}$ = 0.012] on number of risky choices made in the reflection trials.

We did not observe any significant correlations between CD symptoms and risk preferences on the 8 ΔEV trials or between CD symptoms and risk preferences on the reflection trials.

### Potential confounding factors

When controlling for IQ differences between the groups, the results for the primary analysis of the 8 ΔEV trial types were unchanged, i.e., significant main effects of Group [*F*(1, 95) = 12.10, *p* = 0.001; $\eta_{\text{p}}^{2}$ = 0.113], and Trial Type [*F*(4.24, 402.96) = 2.06, *p* = 0.045; $\eta_{\text{p}}^{2}$ = 0.021], as well as a significant Trial Type × Group interaction [*F*(4.24, 402.96) = 3.13, *p* = 0.013, $\eta_{\text{p}}^{2}$ = 0.032] and a trend towards a Group × Sex interaction [*F*(1, 95) = 3.04, *p* = 0.084; $\eta_{\text{p}}^{2}$ = 0.031; observed power = 0.408] were still observed. The results for the reflection trials also remained the same, i.e., there was still a main effect of Condition [*F*(1, 95) = 9.84, *p* = 0.002; $\eta_{\text{p}}^{2}$ = 0.094], but no other significant main effects or interactions.

When controlling for ADHD symptoms, the results on the 8 ΔEV trial types remained largely unchanged. There was a main effect of Condition [*F*(4.23, 402.15) = 207.13, *p* < 0.001; $\eta_{\text{p}}^{2}$ = 0.686] and Group [*F*(1, 95) = 9.97, *p* = 0.002; $\eta_{\text{p}}^{2}$ = 0.095] and a trend towards a Group × Sex interaction [*F*(1, 95) = 3.16, *p* = 0.078; $\eta_{\text{p}}^{2}$ = 0.032; observed power = 0.421]. However, the formerly significant Trial Type × Group interaction was reduced to a trend-level effect [*F*(4.23, 402.15) = 2.02, *p* = 0.086; $\eta_{\text{p}}^{2}$ = 0.021; observed power = 0.642]. When considering the reflection trials, all the results remained unchanged, i.e., there was only a significant main effect of Condition [*F*(1, 95) = 152.42, *p* < 0.001; $\eta_{\text{p}}^{2}$ = 0.616], but no other main effects or interactions.

## Discussion

The present study investigated sex differences in risky decision making in individuals with CD. There were several findings of note. First, as predicted, we found that adolescents with CD displayed elevated levels of risk taking—an effect that was most evident on the trial types where the two expected values were relatively similar, and thus which of the options was most favourable was not as clear. Overall, the participants were highly sensitive to the differences in expected value between the trial types, showing a robust sigmoidal function from the most unfavourable to the most favourable trial types. Second, in line with our predictions, a trend towards a Group × Sex interaction effect was observed, indicating that increased risk taking was more evident in males with CD than their female counterparts, relative to their respective sex-matched control groups. Again, this effect was most evident in the mid-range of expected values, where the favourable option was less apparent. Conversely, on trials where the experimental gamble was either highly favourable (EV +55) or unfavourable (EV −55), choice of the risky option approached ceiling or floor levels, respectively, which likely restricted our ability to detect group differences or demonstrate impairments in risk taking on these trial types. Third, we showed that the framing of the trial in terms of gains or losses dramatically influenced the participants’ tendency to select the risky choice—all participants were more likely to select the risky gamble in the ‘loss’ than the ‘gain’ condition, consistent with the classic reflection effect, but this bias did not vary by either group or sex.

### Decision making as a function of expected value

Importantly, the CD group showed a general propensity to select the risky option more frequently than the typically developing group—consistent with the existing literature which has predominantly focused on males [8, 11, 13, 31, 42]. Moreover, the heightened propensity towards risk taking in the CD group was most evident on the trials that are most sensitive to individual differences in risk preferences (see, Fig. 2), i.e., trial types in which ceiling or floor effects were not seen. Crucially, when controlling for the potentially confounding effects of ADHD symptoms and IQ differences, the initial results remained largely unchanged—only the Trial Type × Group interaction was reduced to a trend-level finding when accounting for ADHD symptoms. Hence, our current findings are consistent with the idea that alterations in decision making processes in CD are related to altered reward or punishment sensitivity. These alterations may relate to abnormalities in the initial value appraisal of choice alternatives and could potentially lead to the reckless behaviours typically seen in individuals with CD—they may disregard the potential harmful consequences of dangerous or risky actions. From a neural perspective, these abnormalities are thought to be related to functional disturbances or structural deficits in brain regions implicated in reinforcement processing, such as the orbitofrontal cortex, amygdala, anterior cingulate, ventral striatum and insula [2, 11].

A trend towards a Group × Sex interaction effect, which suggested that CD males exhibited a propensity towards significantly greater risk preferences in comparison to typically developing males, while in contrast CD females tended to show risk preferences comparable to those of healthy females, is the most critical and novel finding of this study. This implies that the relationship between CD and risky decision making may differ by sex. Hence, on the one hand, our current results are in line with the previous studies employing male-only samples, indicating an increased tendency for risk-seeking behaviours in males with CD [8, 13, 31]. On the other hand, the results that were obtained when testing for interactions between group and sex in the current study tentatively suggest that males and females with CD have different neurocognitive profiles in relation to risky decision making and risk preferences. On the basis of previous research, it has been argued that increased risk taking may represent an altered balance between emotional and decision making processes [43, 44]. It seems that this imbalance may be expressed more strongly in males with CD. This indication that females with CD may differ from their male counterparts in the processing of rewards and risk taking preferences suggests that there may be different developmental pathways to CD in males and females [45], with abnormalities in reward processing mechanisms potentially more influential in predisposing towards antisocial behaviour in males than females.

The current findings may also indicate a sex-differentiated effect of CD on reward and loss sensitivity, suggesting that males with CD are hypersensitive to the possibility of obtaining rewards or hyposensitive to potential losses. It is possible that other motivational aspects are involved, pointing to a generally increased reward (at any cost)-oriented motivational style in males with CD in particular. When controlling for the potentially confounding effects of ADHD symptoms, the initial results remained largely unchanged—only the Trial Type × Group interaction was reduced to a trend-level finding. Moreover, controlling for group differences in IQ did not alter the results for the main expected value trials.

### Reflection effects

The reflection trials were primarily included to investigate risk-seeking and/or risk aversion, as each gamble has an equal expected value, but one is more risky, i.e., involves a greater degree of outcome variance, than the other. As expected, our data showed that the framing of the trial in terms of a ‘loss’ or a ‘gain’ differentially affected risk-seeking behaviour. The risky gamble was generally selected by our participants in the ‘loss’ condition (in preference to a certain moderate loss), while risk aversion was more evident in the ‘gain’ condition—a well-established finding in the normative behavioural economic literature in adults [24, 28, 44]. Importantly, controlling for group differences in ADHD symptoms and IQ did not alter the initial findings related to reflection effects. The fact that we did not observe any effects of Group, Sex or Group-by-Sex interactions on choices made on these trials, suggests that risk-seeking and risk aversion preferences as a function of the framing of a situation are unaffected in individuals with CD.

### Limitations

These results should be interpreted in light of several limitations. In particular, our ability to test for Sex-by-Group interactions was limited by a moderate sample size (*N* = 100). That said, our trend-level interaction was robust to controlling for IQ and ADHD symptoms, and simple effects analysis yielded a statistically significant group difference in males, with a large effect size ($\eta_{\text{p}}^{2}$ = 0.278), combined with a non-significant difference with a small effect size in females ($\eta_{\text{p}}^{2}$ = 0.029). Another possible limiting factor is the heterogeneity of CD. Different clusters of symptoms potentially lead to the same CD diagnosis and there also appear to be differences between the sexes in vulnerability to comorbid disorders [46]. It could be hypothesized that girls with CD tend to be less risk taking than their male counterparts because of higher levels of comorbid anxiety or mood disorders in the former group; however, this explanation is unlikely, at least in the current sample, since none of CD subjects had comorbid generalised anxiety disorder. In addition, it would have been interesting to test for the potential sex differences in the relationship between callous-unemotional (CU) personality traits and risky decision making in the CD group. CU traits are a downward extension of the psychopathy concept to children, and map closely onto the affective dimension of psychopathy [47]. Although previous studies have reported that CU traits do not significantly modulate risk taking in males with CD [8, 31, 48], the relationship between CU traits and risk taking in CD females is unknown. However, in the current study we were unable to reliably address this issue because of the limited variance in the CU traits measure and the small sample size. Lastly, future studies should employ more refined experimental paradigms that allow decomposition of different stages of the decision making process (choice, execution, and outcome processing), which would enable researchers to examine whether, and how, they are affected in individuals with CD [2].

### Clinical implications

From a clinical perspective, if the current results of potential sex differences in the relationship between CD and risk taking are confirmed in follow-up research, it would add to the existing, although limited, literature on sex differences in the manifestation of CD as well as deepening our understanding of the mechanisms involved in risky decision making and their differentiation between the sexes in CD. This could in turn aid the diagnosis and assessment of CD, as well as help improve existing treatments and facilitate the development of novel intervention strategies.

## Conclusions

In the present study, we investigated risky decision making in adolescents with CD, and also examined whether the relationship between CD and decision making differs by sex. Consistent with previous research, our results show that adolescents with CD in general tend to be hypersensitive to potential gains and less sensitive to potential losses compared with typically developing adolescents. In terms of sex differences, our data suggested a trend towards a potential sex difference in the relationship between CD diagnosis and risk taking propensity. While males with CD showed excessive risk-seeking relative to typically developing males, females with CD in contrast appeared to be more cautious and did not differ from typically developing females in terms of their decision making behaviour. Our current results, although preliminary and in need of replication, are broadly consistent with the idea that there may be different developmental pathways and causal mechanisms leading to CD in males and females. However, we note that larger-scale and particularly longitudinal studies investigating risk and reward processing in males and females at risk for developing CD are needed to adequately test this hypothesis.
